# Diagnostic Performance of Serum Leucine-Rich Alpha-2-Glycoprotein 1 and Plasma Calprotectin in Adults with Suspected Acute Appendicitis: A Cross-Sectional Observational Study

**DOI:** 10.3390/biomedicines14092022

**Published:** 2026-09-08

**Authors:** Lea Maršić, Zvjezdana Gvozdanović, Nikolina Farčić, Luka Maršić, Željka Dujmić, Željka Dragila Tomašić, Denis Klapan, Dino Vida, Josip Samardžić, Vikica Buljanović, Dag Dušanić, Andrea Saračević, Helena Čičak, Zrinka Mihaljević, Višnja Nesek Adam

**Affiliations:** 1 Department of Emergency Medicine, General Hospital Našice, 31500 Našice, Croatia; gvozdanovic.lea@gmail.com (L.M.); klapandenis@gmail.com (D.K.); 2 Faculty of Medicine Osijek, Josip Juraj Strossmayer University of Osijek, 31000 Osijek, Croatia; zeljkadragila5@gmail.com (Ž.D.T.); vikica.buljanovic@gmail.com (V.B.); 3 Faculty of Dental Medicine and Health Osijek, Josip Juraj Strossmayer University of Osijek, 31000 Osijek, Croatia; zg9598@gmail.com (Z.G.); nikfarcic@gmail.com (N.F.); dujmic.zeljka@gmail.com (Ž.D.); josip.samardzic@gmail.com (J.S.); 4 Department of Nursing, University of Applied Sciences in Bjelovar, 43000 Bjelovar, Croatia; 5 Department of Emergency Medicine, General Hospital “Dr. Josip Benčević” Slavonski Brod, 35000 Slavonski Brod, Croatia; luka.marsic@gmail.com; 6 General Hospital “Dr. Josip Benčević” Slavonski Brod, 35000 Slavonski Brod, Croatia; 7 Department of Emergency Medicine, University Hospital Centre Osijek, 31000 Osijek, Croatia; 8 General Hospital Našice, 31500 Našice, Croatia; dino.vida@gmail.com; 9 Business Economics and Sustainable Development, Libertas International University, 10000 Zagreb, Croatia; 10 Department of Abdominal Surgery, General Hospital “Dr. Josip Benčević” Slavonski Brod, 35000 Slavonski Brod, Croatia; 11 Medical Biochemistry Laboratory, General Hospital Našice, 31500 Našice, Croatia; 12 Department of Pathology, General Hospital Našice, 31500 Našice, Croatia; dagdusanic@gmail.com; 13 Department of Medical Laboratory Diagnostics, University Hospital “Sveti Duh”, 10000 Zagreb, Croatia; asaracevic@kbsd.hr (A.S.); helena.cicak5@gmail.com (H.Č.); 14 Department of Physiology and Immunology, Faculty of Medicine Osijek, Josip Juraj Strossmayer University of Osijek, 31000 Osijek, Croatia; 15 Department of Anesthesiology, University Hospital “Sveti Duh”, 10000 Zagreb, Croatia; 16 Department of Emergency Medicine, University Hospital “Sveti Duh”, 10000 Zagreb, Croatia

**Keywords:** acute appendicitis, biomarkers, calprotectin, leucine-rich alpha-2-glycoprotein 1, LRG1

## Abstract

**Background/Objectives:** This study evaluated the diagnostic performance of serum leucine-rich alpha-2-glycoprotein 1 (LRG1) and plasma calprotectin in adults with suspected acute appendicitis, compared them with routine inflammatory markers and clinical scores, and examined their association with abdominal guarding and the discrimination of uncomplicated from complicated appendicitis. **Methods:** Adults aged ≥18 years were enrolled in two Croatian emergency departments from April 2025 to March 2026. Acute appendicitis was histopathologically confirmed, whereas controls were defined by clinical follow-up or negative appendiceal histopathology. Biomarkers were measured using enzyme-linked immunosorbent assays, and diagnostic performance was assessed using receiver operating characteristic (ROC) analysis with area under the curve (AUC). **Results:** Among 126 patients, 62 (49.2%) had acute appendicitis and 64 (50.8%) were controls. Serum LRG1 was higher in patients with acute appendicitis than controls (27.4 μg/mL; IQR 25.3–28.0 vs. 23.2 μg/mL; IQR 17.6–27.8; *p* = 0.01), whereas plasma calprotectin did not differ. LRG1 was higher in cases with abdominal guarding (*p* = 0.02), but neither biomarker differed significantly between uncomplicated and complicated appendicitis. **Conclusions:** LRG1 showed limited diagnostic accuracy, while calprotectin showed no meaningful discrimination. The Adult Appendicitis Score and Appendicitis Inflammatory Response score outperformed both biomarkers.

## 1. Introduction

Acute appendicitis is one of the most common causes of acute abdominal pain requiring urgent surgical evaluation, yet its diagnosis remains challenging, particularly during the early stages of the disease [1]. Despite advances in diagnostic strategies, negative appendectomy rates remain between 15 and 40%, while perforation rates range from 15 to 30% [2,3,4,5,6,7].

Historically a clinical diagnosis, acute appendicitis is now diagnosed using a multimodal approach that combines clinical evaluation, laboratory testing, clinical scoring systems, and imaging studies, although adherence to recommended diagnostic and management pathways remains variable in clinical practice [8,9]. Early recognition is difficult, as the goal is to identify the condition in its initial stage, when inflammatory changes may not be clearly reflected in laboratory findings [10,11]. Diagnostic uncertainty is further increased by atypical clinical presentations and the broad differential diagnosis of acute abdominal pain [12,13,14]. Although white blood cell (WBC) count and C-reactive protein (CRP) are routinely used in diagnostic workup of acute appendicitis, their diagnostic performance varies considerably across studies [15,16,17,18,19]. Clinical scoring systems, including the Appendicitis Inflammatory Response (AIR) score and Adult Appendicitis Score (AAS), improve diagnostic performance by integrating clinical and laboratory findings and are recommended in the latest World Society of Emergency Surgery (WSES) Jerusalem guidelines for risk stratification in patients with suspected acute appendicitis [8]. While they may help identify low-risk patients suitable for outpatient follow-up, a substantial proportion of patients fall into the intermediate-risk category, where diagnostic uncertainty persists and further investigations are often required [20,21]. In this setting, imaging modalities, particularly ultrasonography and computed tomography (CT), improve diagnostic certainty but have important limitations, including operator dependency, radiation exposure, cost, and limited availability in some healthcare settings [22,23,24]. Distinguishing uncomplicated from complicated appendicitis is clinically important because it influences treatment strategy, including consideration of nonoperative management in selected uncomplicated cases [8,25]. Uncomplicated appendicitis is characterized by inflammation without necrosis, perforation, or abscess, whereas complicated appendicitis includes gangrene, perforation, or periappendiceal abscess. However, reliable differentiation remains challenging despite combined clinical, laboratory, and imaging assessment [25]. Consequently, diagnostic uncertainty contributes to both unnecessary surgical interventions and delayed treatment. Despite these advances, a significant gap remains the absence of a simple, reliable, and widely accessible biomarker that could support early clinical decision-making.

Over the past decade, leucine-rich alpha-2-glycoprotein 1 (LRG1) and calprotectin have emerged as increasingly studied inflammatory biomarkers [26,27]. However, most available evidence comes from pediatric studies, and their diagnostic value in adult patients with acute appendicitis has not been clearly established [28]. LRG1 is an acute-phase protein involved in immune and inflammatory responses, as well as in certain pathological conditions such as infections and malignancies [29]. While its precise physiological function remains incompletely defined, LRG1 is thought to contribute to intercellular signaling and tissue remodeling [30]. It is produced by various cell types, including neutrophils, hepatocytes, and endothelial cells [31]. Increased LRG1 levels have been reported in patients with acute appendicitis in blood, urine, and saliva, suggesting that it may reflect the underlying inflammatory response and, potentially, disease severity [32,33,34,35]. Calprotectin (S100A8/A9) is a protein complex composed of two calcium-binding subunits that belong to the S100 family. As the most abundant cytosolic protein in neutrophils, calprotectin is released during inflammatory processes and participates in immune regulation by promoting WBC recruitment and amplifying the inflammatory response [36,37]. Its concentration increases with neutrophil migration into affected tissues, particularly in the gastrointestinal tract, which led to its widespread use as a biomarker of intestinal inflammation [38,39]. Given its established role in intestinal inflammatory processes, a similar increase could be expected in acute appendicitis. Consistent with this assumption, several studies have reported elevated calprotectin concentrations in patients with acute appendicitis, although its diagnostic performance has varied across studies [40,41,42,43,44].

This study aimed to evaluate the diagnostic accuracy of serum LRG1 and plasma calprotectin in adults presenting with suspected acute appendicitis, including comparison with routinely used inflammatory markers and clinical scoring systems. Secondary objectives were to assess their association with abdominal guarding and their ability to distinguish uncomplicated from complicated appendicitis among patients with confirmed acute appendicitis.

## 2. Materials and Methods

### 2.1. Study Design

This was a cross-sectional observational study designed to evaluate the diagnostic accuracy of serum LRG1 and plasma calprotectin in adults presenting with suspected acute appendicitis. The study protocol was approved by the Ethics Committees of General Hospital Našice, University Hospital “Sveti Duh” in Zagreb, and the Faculty of Medicine Osijek (approval numbers: 01-505/2-2022, 03-7225, and 2158-61-46-23-152, respectively). All participants received verbal and written information about the study, and written informed consent was obtained before enrollment. The study was reported in accordance with the STARD (Standards for Reporting Diagnostic Accuracy Studies) statement.

### 2.2. Study Setting and Population

The study was conducted from April 2025 to March 2026 in the emergency departments (EDs) of two Croatian hospitals: General Hospital Našice, a secondary care hospital, and University Hospital “Sveti Duh”, Zagreb, a tertiary care hospital. Adult patients presenting with suspected acute appendicitis were consecutively screened for eligibility.

The inclusion criteria were age ≥ 18 years, localized right lower quadrant abdominal pain, and symptoms suggestive of acute appendicitis, such as initial periumbilical pain, pain migration, nausea and/or vomiting, fever, or anorexia. The exclusion criteria were inflammatory bowel disease, known active malignancy, autoimmune disease, immunosuppressive therapy, pregnancy, previous appendectomy, psychiatric disorder, obesity (body mass index (BMI) ≥ 30 kg/m^2^), antibiotic use before ED presentation, and recent abdominal trauma. The two patients with colorectal cancer reported in the control group had no known malignancy at enrollment and were diagnosed during the index evaluation. Therefore, they did not meet this exclusion criterion. Patients with obesity and those who had received antibiotics before ED presentation were excluded to reduce potential confounding of inflammatory biomarker concentrations by chronic low-grade inflammation and treatment-related modification of the acute inflammatory response, respectively.

Patients were classified into two groups: patients with histopathologically confirmed acute appendicitis and controls in whom acute appendicitis was excluded based on clinical assessment and follow-up or, when applicable, negative appendiceal histopathology. All controls discharged from the ED and managed nonoperatively (*n* = 41) were contacted by telephone 48 h after discharge. None had a subsequent ED presentation, hospitalization, or appendectomy during the 48 h follow-up period. Long-term follow-up was not performed.

### 2.3. Study Protocol

As part of routine evaluation of patients with suspected acute appendicitis, demographic data, including age and sex, and clinical symptoms were recorded at presentation. Clinical variables included symptom duration, nausea and/or vomiting, anorexia, pain migration, body temperature, and abdominal guarding. Duration of symptoms was defined as the time from patient-reported symptom onset to ED presentation. Venous blood samples were obtained for routine laboratory testing, including WBC count, absolute neutrophil count (ANC) and neutrophil percentage, CRP, procalcitonin, mean platelet volume (MPV), neutrophil-to-lymphocyte ratio (NLR), and platelet-to-lymphocyte ratio (PLR). Additional venous blood samples were collected for serum LRG1 and plasma calprotectin measurement. For each participant, the Alvarado score, AIR, and AAS were calculated using clinical and laboratory data obtained at presentation.

Imaging was performed as part of routine clinical care at the discretion of the treating physician and was not mandated by the study protocol. Ultrasonography and/or CT findings, when available, were considered together with clinical and laboratory findings to guide patient management, while some patients proceeded to surgery based on clinical assessment without additional imaging.

### 2.4. Biomarker Sampling and Measurement

For serum LRG1 measurement, venous blood was collected in a 3.5 mL serum collection tube, while for plasma calprotectin measurement, venous blood was collected in a 2 mL tube containing ethylenediaminetetraacetic acid (EDTA). Blood samples were transported to the laboratory immediately after collection and centrifuged without delay. However, the exact collection-to-centrifugation time was not recorded. Serum samples were centrifuged for 15 min at 1000× *g*, and plasma samples for 20 min at 2000× *g*. At least two aliquots of ≥300 µL were prepared from each sample in 1.5 mL microcentrifuge tubes. All serum and plasma aliquots were labeled with a unique study identification code, sampling date, and sample type, and were stored at −80 °C until batch analysis after completion of patient recruitment. After transport on dry ice to the Laboratory for Molecular and Clinical Immunology, Faculty of Medicine Osijek, samples were thawed for ELISA analysis and underwent a single freeze–thaw cycle. Biomarker analyses were performed on the same day.

Serum LRG1 concentrations were measured using a commercially available ELISA kit (MyBioSource, San Diego, CA, USA; MBS701497) and expressed in µg/mL. The assay detection range was 0.156–40 µg/mL. The manufacturer-reported intra-assay and inter-assay coefficients of variation were <12% and <15%, respectively. Plasma calprotectin concentrations were measured using a commercially available ELISA kit (BT LAB, Jiaxing, China; E4010Hu) and expressed in ng/mL. The standard curve range was 2–600 ng/mL. The manufacturer-reported intra-assay and inter-assay coefficients of variation were <8% and <10%, respectively. Absorbance for both ELISA assays was measured at 450 nm using a compact 96-well microplate reader (PR 3100 TSC, Bio-Rad Laboratories, Hercules, CA, USA). Both assays were performed according to the manufacturer’s instructions, and all samples were analyzed in duplicate. All LRG1 and calprotectin concentrations were within the respective assay measurement ranges, and no values required censoring, imputation, or exclusion. Laboratory personnel performing the biomarker analyses were blinded to the final diagnosis, clinical scores, and routine laboratory results.

### 2.5. Histopathologic Assessment

In patients who underwent surgery, the removed appendix was submitted for standard histopathologic examination at the pathology departments of the respective hospitals. Laboratory personnel performing LRG1 and calprotectin measurements were blinded to the final diagnosis and clinical scoring results. Histopathological assessment was performed as part of routine care, and biomarker results were not available to the pathologists at the time of assessment. Histopathologic findings were classified as positive or negative for acute appendicitis. Positive findings were further categorized as uncomplicated or complicated acute appendicitis. Uncomplicated acute appendicitis was defined as phlegmonous or suppurative inflammation without necrosis, perforation, or abscess. Complicated acute appendicitis was defined as gangrenous or perforated appendicitis or the presence of a periappendiceal abscess.

### 2.6. Sample Size Calculation

Sample size was determined a priori using G*Power version 3.1.2. The original calculation was based on detecting a medium between-group effect in biomarker concentrations, with a two-sided α level of 0.05 and statistical power of 0.90, resulting in a required sample size of 125 participants. A separate calculation for correlation analyses, assuming a medium effect size and 0.85 power, required 105 participants. At the time of study planning, sufficiently robust adult data were not available to prespecify an expected area under the curve (AUC) for LRG1 or calprotectin. Therefore, the sample size was not based specifically on receiver operating characteristic (ROC) analysis. The planned minimum sample size was 125 participants.

### 2.7. Statistical Analysis

Categorical variables are presented as counts and percentages and were compared using the chi-square test. Continuous variables were assessed for normality using the Shapiro–Wilk test and are presented as median and interquartile range (IQR). Between-group comparisons were performed using the Mann–Whitney U test. Associations between variables were assessed using Spearman rank correlation coefficients. No indeterminate index test or reference standard results were observed, and all patients included in the final analysis had complete clinical, laboratory, biomarker, and reference standard data.

Diagnostic performance was assessed using ROC curve analysis, including calculation of the AUC, sensitivity, specificity, and optimal cutoff values. Optimal cutoff values were determined exploratorily using the Youden index. Differences between AUCs were compared using the DeLong test. Factors associated with acute appendicitis were evaluated using bivariable and multivariable logistic regression analyses. All tests were two-sided, and *p* values < 0.05 were considered statistically significant. Statistical analyses were performed using MedCalc Statistical Software version 23.4.8 (MedCalc Software Ltd., Ostend, Belgium).

## 3. Results

### 3.1. Patient Characteristics

**Figure 1:**
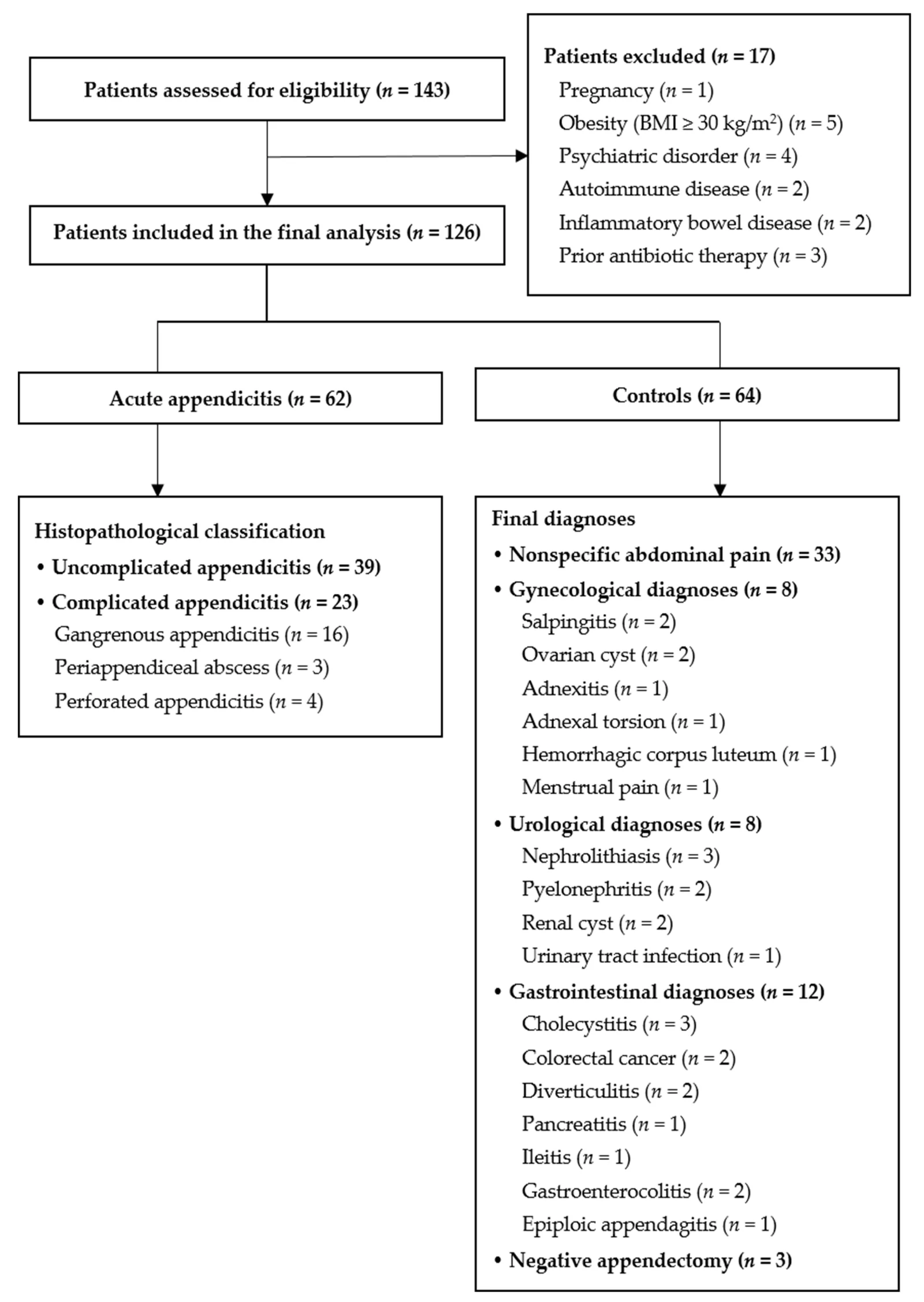
Flowchart of patient inclusion and final diagnoses. BMI = body mass index.

Of 143 patients with suspected acute appendicitis assessed for eligibility, 126 were included in the final analysis after 17 exclusions (Figure 1). The study population included 71 male (56.3%) and 55 female (43.7%) patients, with a median age of 37 years (range, 18–83 years). Acute appendicitis was histopathologically confirmed in 62 (49.2%) patients, while 64 (50.8%) were controls. Sex and age distribution did not differ significantly between groups. Final diagnoses among patients in the control group are summarized in Table 1. Perforated appendicitis was observed in 4 patients (3.2%), and the negative appendectomy rate among patients undergoing appendectomy was 4.6% (3/65), with all three cases occurring in women. Imaging was performed in 48 patients (38.1%), of whom 23 underwent ultrasonography only, 20 underwent CT only, and 5 underwent both modalities.

### 3.2. Primary Outcome Measures

Serum LRG1 concentrations were significantly higher in patients with acute appendicitis than in controls (27.4 μg/mL; IQR 25.3–28.0 vs. 23.2 μg/mL; IQR 17.6–27.8; *p* = 0.01). In contrast, plasma calprotectin did not differ significantly between groups (126.7 ng/mL; IQR 89.3–195.6 vs. 121.9 ng/mL; IQR 90.9–203.4; *p* = 0.82).

**Figure 2:**
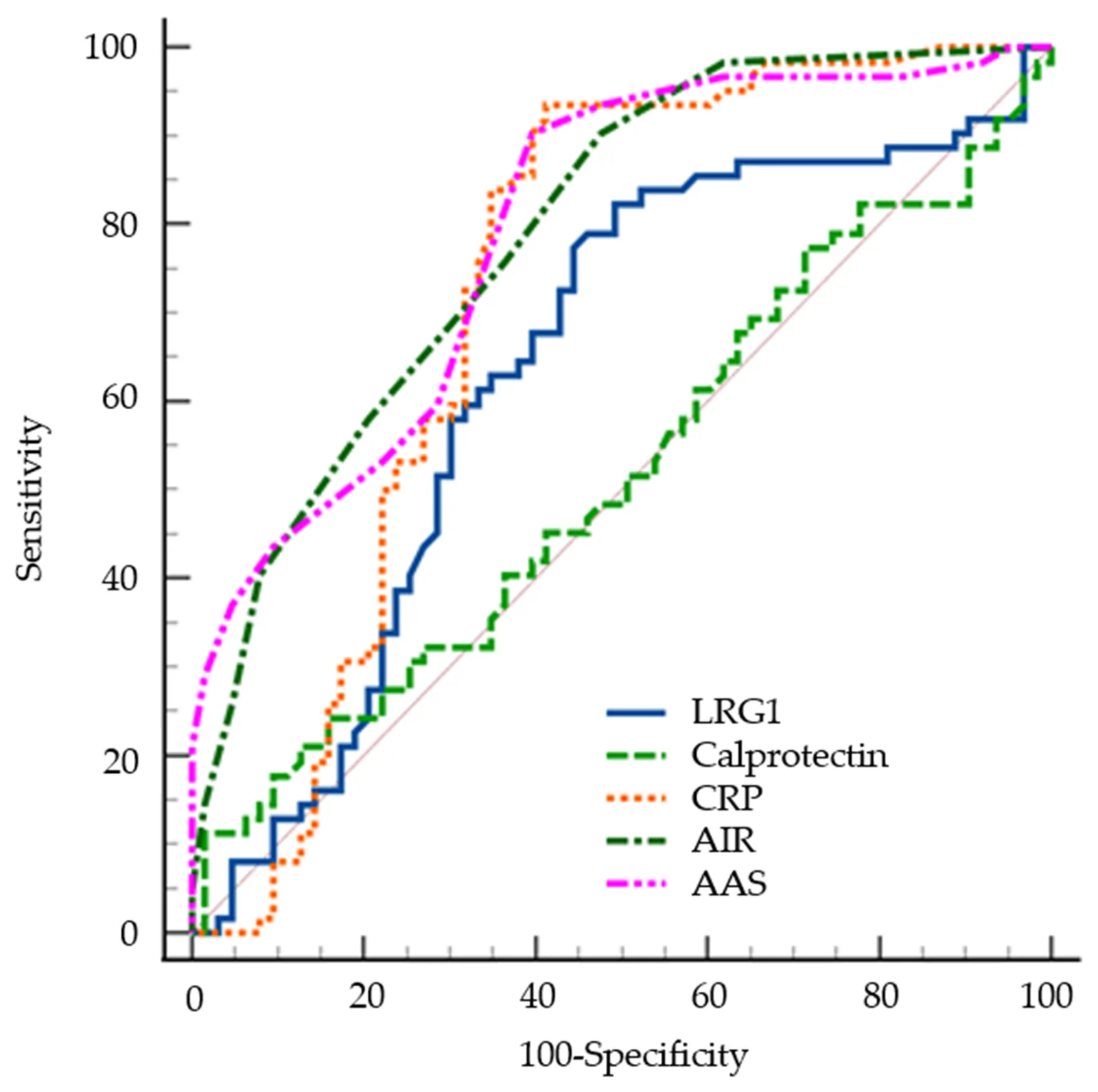
Receiver operating characteristic curves for LRG1, calprotectin, CRP, AIR and AAS. LRG1 = leucine-rich alpha-2 glycoprotein 1; CRP = C-reactive protein; AIR = appendicitis inflammatory response score; AAS = adult appendicitis score.

In ROC analysis, serum LRG1 demonstrated limited diagnostic accuracy for acute appendicitis (AUC = 0.630; 95% CI, 0.540–0.715), with a sensitivity of 82.3% and specificity of 50.0% at the optimal cutoff value. Plasma calprotectin showed no meaningful discriminative ability (AUC = 0.512; 95% CI, 0.421–0.602). Among the laboratory markers, CRP showed the highest diagnostic accuracy (AUC = 0.727), while the AIR and AAS scores demonstrated the best overall performance (AUCs of 0.795 and 0.798, respectively) (Table 2). ROC curves for LRG1, calprotectin, CRP, AIR and AAS are shown in Figure 2.

DeLong’s test showed that ANC and neutrophil percentage had diagnostic performance comparable to LRG1 but significantly higher than calprotectin. AIR and AAS had significantly higher AUCs than LRG1. Adding LRG1 to CRP, AIR, or AAS did not significantly improve diagnostic discrimination (Table 3).

### 3.3. Secondary Outcome Measures

Compared to controls, patients with acute appendicitis more frequently had pain migration, fever, and abdominal guarding (Table 4). Among patients with acute appendicitis, the median duration of symptoms before ED presentation was 24 h (IQR, 12–48 h).

Patients with acute appendicitis had higher WBC count, CRP, procalcitonin, ANC, neutrophil percentage, and NLR than controls. Other laboratory findings did not differ significantly between groups (Table 5).

Clinical scoring systems differed significantly between groups, with higher Alvarado, AIR, and AAS scores in patients with acute appendicitis than in controls (all *p* < 0.001). Patients with acute appendicitis were more frequently classified as having intermediate or high risk, whereas controls were more commonly classified as low risk (all *p* < 0.001). High-risk classification was observed in 25 patients (40%) according to the Alvarado score, 3 patients (5%) according to the AIR score, and 15 patients (24%) according to the AAS.

LRG1 showed weak-to-moderate positive correlations with AIR score and inflammatory markers, including CRP, NLR and PLR, whereas calprotectin showed no consistent association with clinical scores or laboratory markers. In bivariable logistic regression, LRG1 was associated with higher odds of acute appendicitis (OR 1.13; *p* = 0.01), whereas calprotectin was not associated with the diagnosis. In multivariable models, the association between LRG1 and acute appendicitis weakened after inclusion of the AIR score, while AIR remained independently associated with the diagnosis (OR 1.98, 95% CI 1.40–2.81; *p* < 0.001). In the model including AAS, both AAS (OR 1.45, 95% CI 1.27–1.72; *p* < 0.001) and LRG1 (OR 1.10, 95% CI 1.01–1.20; *p* = 0.03) remained associated with acute appendicitis.

Among patients with confirmed acute appendicitis, serum LRG1 levels were significantly higher in those with abdominal guarding (median 27.8 μg/mL (IQR, 25.9–28.0) vs. 26.15 μg/mL (IQR, 17.9–27.18); *p* = 0.02), whereas plasma calprotectin did not differ according to the presence of abdominal guarding. No significant differences in LRG1 or calprotectin levels were observed between uncomplicated and complicated appendicitis.

## 4. Discussion

The primary finding of this study was that serum LRG1 was associated with acute appendicitis but demonstrated only modest diagnostic accuracy, whereas plasma calprotectin showed no meaningful discriminative ability. LRG1 correlated with CRP and the AIR score, and was associated with abdominal guarding. However, its low specificity and limited incremental diagnostic value suggest that LRG1 primarily reflects systemic inflammatory activation rather than providing substantial additional diagnostic information.

Previous studies evaluating LRG1 in acute appendicitis have reported inconsistent results. Most available evidence originates from pediatric populations, in which LRG1 has generally demonstrated moderate-to-excellent diagnostic performance. Reported AUC values range from 0.69 to 1.00, generally exceeding the AUC observed in our study [32,45,46,47,48]. In adults, Rainer et al. reported an AUC of 0.742 for plasma LRG1, whereas a combined model incorporating LRG1, LRG1 mRNA, and the Alvarado score achieved an AUC of 0.845 [49]. In contrast, Lontra et al. found no significant difference in plasma LRG1 concentrations between adults with and without acute appendicitis [50]. These discrepancies likely reflect differences in study design, patient selection, sample type, analytical methods, case definition, and diagnostic thresholds.

Unlike LRG1, plasma calprotectin did not discriminate between patients with and without acute appendicitis. Although calprotectin demonstrated high specificity at the selected cutoff, its very low sensitivity would result in a substantial proportion of missed cases, limiting its clinical utility as a diagnostic marker. This finding contrasts with several previous studies reporting favorable diagnostic performance of plasma and serum calprotectin, including AUC values exceeding 0.90 [44,51,52]. However, other studies reported considerably lower diagnostic accuracy, with AUC values ranging from 0.58 to 0.67 and poor specificity [42,43]. The marked heterogeneity across studies suggests that the diagnostic performance of calprotectin is highly dependent on patient characteristics, sample type, assay methodology, and study design [53].

The neutrophil-associated nature of LRG1 and calprotectin should also be considered in the broader context of neutrophil activation in acute appendicitis. Previous studies have evaluated markers of neutrophil activation and neutrophil extracellular trap (NET) formation, including cell-free DNA (cfDNA), myeloperoxidase (MPO), neutrophil elastase (NE), and citrullinated histone H3 (H3Cit) [54,55]. These markers were elevated in appendicitis and associated with disease severity, while cfDNA and H3Cit demonstrated promising diagnostic performance in pediatric patients, with H3Cit also distinguishing uncomplicated from complicated appendicitis [55]. These findings suggest that markers of neutrophil activation and NET formation warrant further investigation as potential diagnostic and severity-related biomarkers in acute appendicitis.

A clinically important finding of this study was the superior performance of established clinical scoring systems. Both the AIR and AAS achieved higher diagnostic accuracy than either biomarker and were significantly more accurate than LRG1. Furthermore, adding LRG1 to CRP, AIR, or AAS did not significantly improve diagnostic discrimination, indicating limited incremental diagnostic value beyond routinely available inflammatory markers and established clinical scoring systems. The association between LRG1 and acute appendicitis also weakened after adjustment for clinical scoring systems, suggesting that much of the information captured by LRG1 overlaps with routinely available clinical and laboratory variables already incorporated into these scores.

Strengths of this study include consecutive patient recruitment, a two-center setting, histopathological confirmation of surgically treated cases, clinical follow-up of nonoperatively managed controls, and the comparison of novel biomarkers with routine inflammatory markers and established clinical scoring systems in an adult population, which remains underrepresented in the current literature. Several limitations should be noted. The sample size was based on between-group biomarker differences rather than a prespecified diagnostic-accuracy parameter such as AUC, which may limit the precision of the ROC estimates. The relatively small number of patients with complicated appendicitis, particularly perforated cases, limited the statistical power to detect biomarker differences according to disease severity. Therefore, the absence of significant differences should not be interpreted as evidence of no association, and these findings should be considered exploratory. The control group consisted of clinically diverse adults in whom appendicitis was ultimately excluded, rather than healthy individuals. They may have had different inflammatory and non-inflammatory causes of abdominal pain. This heterogeneity may have reduced biomarker specificity and attenuated between-group differences, although it reflects the real-world diagnostic challenge in the ED. Although all controls discharged from the ED were contacted 48 h after discharge and none reported a subsequent ED presentation, hospitalization, or appendectomy during this period, long-term follow-up was not performed. Therefore, delayed or initially missed appendicitis cannot be completely excluded, introducing a potential risk of disease misclassification and differential verification bias. The exclusion of patients with obesity and prior antibiotic use may also limit the generalizability of our findings to the broader population encountered in routine clinical practice. Biomarkers were measured at a single point, precluding assessment of temporal changes. In addition, only serum LRG1 and plasma calprotectin were evaluated, and differences in sample type, ELISA assays, and laboratory procedures may limit direct comparison with previous studies. Symptom duration before sampling varied among patients and was based on patient history, which may have influenced biomarker concentrations. Finally, standardized cutoff values for LRG1 and calprotectin in acute appendicitis are lacking, limiting immediate clinical interpretation. Further studies should include larger adult cohorts, serial biomarker measurements, and validation of clinically useful cutoff values.

## 5. Conclusions

In adults with suspected acute appendicitis, serum LRG1 showed limited diagnostic accuracy and low specificity, although it outperformed plasma calprotectin. Plasma calprotectin showed no meaningful discriminative ability. Higher LRG1 levels in patients with abdominal guarding suggest an association with inflammatory burden. In an exploratory analysis, no significant differences in either biomarker were observed between uncomplicated and complicated appendicitis. Established clinical scoring systems, particularly AIR and AAS, provided superior diagnostic performance, while the potential role of LRG1 as an adjunctive biomarker requires further evaluation.

## Figures and Tables

**Table 1:** Final diagnoses in the control group (*n* = 64).

Final Diagnosis	*n* (%) *
Nonspecific abdominal pain	33 (51.6)
Gynecological diagnoses	8 (12.5)
Salpingitis	2 (3.1)
Ovarian cyst	2 (3.1)
Adnexitis	1 (1.6)
Adnexal torsion	1 (1.6)
Hemorrhagic corpus luteum	1 (1.6)
Menstrual pain	1 (1.6)
Urological diagnoses	8 (12.5)
Nephrolithiasis	3 (4.7)
Pyelonephritis	2 (3.1)
Renal cyst	2 (3.1)
Urinary tract infection	1 (1.6)
Gastrointestinal diagnoses	12 (18.8)
Cholecystitis	3 (4.7)
Colorectal cancer	2 (3.1)
Diverticulitis	2 (3.1)
Pancreatitis	1 (1.6)
Ileitis	1 (1.6)
Gastroenterocolitis	2 (3.1)
Epiploic appendagitis	1 (1.6)
Negative appendectomy	3 (4.7)
Total	64 (100)

* Percentages are calculated within the control group (*n* = 64).

**Table 2:** Diagnostic accuracy of LRG1, calprotectin, laboratory markers, and clinical scores for acute appendicitis.

Variable	Cutoff	Sensitivity, %	Specificity, %	AUC (95% CI)	*p*-Value ^†^
LRG1, μg/mL	>22.6	82.3	50.0	0.630 (0.540–0.715)	**0.01**
CP, ng/mL	>312.8	11.3	98.4	0.512 (0.421–0.602)	0.82
CRP, mg/L	>4.4	93.5	58.7	0.727 (0.640–0.803)	**<0.001**
PCT, μg/L	>0.02	75.8	53.1	0.630 (0.539–0.714)	**0.01**
ANC, ×10^9^/L	>6.86	74.2	60.9	0.723 (0.636–0.799)	**<0.001**
Neutrophils, %	>71	67.7	68.8	0.706 (0.619–0.784)	**<0.001**
NLR	>3.34	70.9	64.1	0.696 (0.608–0.775)	**<0.001**
PLR	>154.2	32.3	81.3	0.549 (0.458–0.638)	0.34
Alvarado	>4	90.3	53.1	0.703 (0.615–0.781)	**<0.001**
AIR	>2	90.3	53.1	0.795 (0.714–0.862)	**<0.001**
AAS	>8	90.3	60.9	0.798 (0.717–0.864)	**<0.001**
LRG1 + CRP	>0.52 *	79.0	55.6	0.637 (0.547–0.722)	**0.007**
LRG1 + AIR	>0.42 *	85.5	67.2	0.800 (0.719–0.866)	**<0.001**
LRG1 + AAS	>0.29 *	93.6	54.7	0.810 (0.731–0.874)	**<0.001**

AUC = area under the curve; CI = confidence interval; LRG1 = leucine-rich alpha-2 glycoprotein 1; CP = calprotectin; CRP = C-reactive protein; PCT = procalcitonin; ANC = absolute neutrophil count; NLR = neutrophil-to-lymphocyte ratio; PLR = platelet-to-lymphocyte ratio; AIR = appendicitis inflammatory response score; AAS = adult appendicitis score. * For combined models, cutoff values refer to predicted probabilities obtained from logistic regression. ^†^ Bold indicates statistical significance (*p* < 0.05).

**Table 3:** Pairwise comparisons of ROC AUCs for investigated biomarkers, laboratory markers, clinical scores, and combined models.

Comparison	AUC Difference (95% CI)	*p*-Value *
Individual predictors vs. investigated biomarkers		
CRP vs. LRG1	0.091 (−0.04 to 0.22)	0.16
PCT vs. LRG1	0.012 (−0.13 to 0.15)	0.86
ANC vs. LRG1	0.092 (−0.039 to 0.223)	0.17
Neutrophil, % vs. LRG1	0.076 (−0.049 to 0.202)	0.24
NLR vs. LRG1	0.057 (−0.07 to 0.19)	0.38
Alvarado vs. LRG1	0.064 (−0.06 to 0.19)	0.32
AIR vs. LRG1	0.165 (0.06 to 0.27)	**0.003**
AAS vs. LRG1	0.158 (0.04 to 0.28)	**0.008**
ANC vs. CP	0.211 (0.087 to 0.334)	**<0.001**
Neutrophils, % vs. CP	0.195 (0.071 to 0.318)	**0.002**
LRG1-based combined models vs. selected predictors		
LRG1 + AIR vs. CRP	0.070 (−0.015 to 0.155)	0.11
LRG1 + AAS vs. CRP	0.081 (−0.009 to 0.172)	0.08
LRG1 + AIR vs. PCT	0.170 (0.069 to 0.271)	**<0.001**
LRG1 + AAS vs. PCT	0.181 (0.08 to 0.28)	**<0.001**
LRG1 + AIR vs. Alvarado	0.097 (0.025 to 0.169)	**0.009**
LRG1 + AAS vs. Alvarado	0.107 (0.031 to 0.183)	**0.006**
Added value of LRG1		
LRG1 + CRP vs. CRP	0.090 (−0.029 to 0.209)	0.14
LRG1 + AIR vs. AIR	0.005 (−0.02 to 0.03)	0.71
LRG1 + AAS vs. AAS	0.012 (−0.02 to 0.04)	0.40

AUC = area under the curve; CI = confidence interval; LRG1 = leucine-rich alpha-2 glycoprotein 1; CRP = C-reactive protein; PCT = procalcitonin; ANC = absolute neutrophil count; NLR = neutrophil-to-lymphocyte ratio; AIR = appendicitis inflammatory response score; AAS = adult appendicitis score; CP = calprotectin. AUC differences were calculated as the first-listed predictor or model minus the second-listed predictor or model. * DeLong test; bold indicates statistical significance (*p* < 0.05).

**Table 4:** Clinical presentation according to final diagnosis.

Clinical Findings	Acute Appendicitis, *n* (%)	Controls, *n* (%)	*p*-Value *
Pain migration	33 (53.2)	22 (34.4)	**0.03**
Fever	19 (30.6)	6 (9.4)	**0.003**
Abdominal guarding	17 (27.4)	1 (1.6)	**<0.001**
Nausea	32 (51.6)	31 (48.4)	0.72
Vomiting	16 (25.8)	16 (25.0)	>0.99
Anorexia	23 (37.1)	29 (45.3)	0.35

* χ^2^ test; bold indicates statistical significance (*p* < 0.05).

**Table 5:** Laboratory findings according to final diagnosis.

Laboratory Marker	Acute Appendicitis	Controls	Difference ^†^	95% CI	*p*-Value *
	Median (IQR)	Median (IQR)			
WBC count, ×10^9^/L	12.3 (9.6–15.0)	9.3 (7.4–11.9)	2.6	1.4 to 4	**<0.001**
CRP, mg/L	34.1 (10.5–66.0)	3.8 (0.7–31.9)	13.6	7.3 to 29.3	**<0.001**
PCT, μg/L	0.040 (0.03–0.07)	0.02 (0.02–0.05)	0.01	0 to 0.02	**0.01**
ANC, ×10^9^/L	9.8 (6.8–12.2)	5.8 (4.4–9.2)	2.8	1.6 to 4.2	**<0.001**
Neutrophil, %	74 (68–74.1)	66 (57–75)	8.7	4.6 to 12.4	**<0.001**
NLR	4.83 (2.9–7.47)	2.87 (1.9–5.32)	1.6	0.76 to 2.58	**<0.001**
PLR	117.3 (100.0–170.2)	113.8 (88–140.2)	7.8	−7.1 to 24.8	0.34
MPV, fL	9.3 (8.6–9.7)	9.2 (8.7–9.7)	0.1	−0.2 to 0.5	0.50

IQR = interquartile range; CI = confidence interval; WBC = white blood cell; CRP = C-reactive protein; PCT = procalcitonin; ANC = absolute neutrophil count; NLR = neutrophil-to-lymphocyte ratio; PLR = platelet-to-lymphocyte ratio; MPV = mean platelet volume. † Hodges-Lehmann estimate of the median difference; * Mann–Whitney U test; bold indicates statistical significance (*p* < 0.05).

## Data Availability

The data that support the findings of this study are available from the corresponding author upon reasonable request. The data are not publicly available due to privacy and ethical restrictions related to patient-level clinical data.

## Abbreviations

The following abbreviations are used in this manuscript:

AAS	Adult Appendicitis Score
AIR	Appendicitis Inflammatory Response
ANC	Absolute Neutrophil Count
AUC	Area Under the Curve
BMI	Body Mass Index
CI	Confidence Interval
cfDNA	cell-free DNA
CP	Calprotectin
CRP	C-Reactive Protein
CT	Computed Tomography
ED	Emergency Department
EDTA	Ethylenediaminetetraacetic Acid
ELISA	Enzyme-Linked Immunosorbent Assay
H3Cit	Citrullinated histone H3
IQR	Interquartile Range
LRG1	Leucine-Rich Alpha-2-Glycoprotein 1
MPO	Myeloperoxidase
MPV	Mean Platelet Volume
NE	Neutrophil Elastase
NET	Neutrophil Extracellular Trap
NLR	Neutrophil-to-Lymphocyte Ratio
OR	Odds Ratio
PCT	Procalcitonin
PLR	Platelet-to-Lymphocyte Ratio
ROC	Receiver Operating Characteristic
STARD	Standards for Reporting Diagnostic Accuracy Studies
WBC	White Blood Cell
WSES	World Society of Emergency Surgery
